# Association between maternal parenting styles and behavioral problems in children with ASD: Moderating effect of maternal autistic traits

**DOI:** 10.3389/fpsyt.2023.1107719

**Published:** 2023-04-18

**Authors:** Xiujin Lin, Xi Su, Saijun Huang, Zhilin Liu, Hong Yu, Xin Wang, Lizi Lin, Muqing Cao, Xiuhong Li, Jin Jing

**Affiliations:** ^1^Department of Maternal and Child Health, School of Public Health, Sun Yat-sen University, Guangzhou, China; ^2^Department of Child Healthcare, Affiliated Foshan Maternity and Child Healthcare Hospital, Southern Medical University, Foshan, China; ^3^The Second School of Clinical Medicine, Southern Medical University, Guangzhou, China; ^4^School of Mathematics and Statistics, Guangdong University of Technology, Guangzhou, China; ^5^Key Laboratory of Brain, Cognition and Education Science, Ministry of Education, China; Institute for Brain Research and Rehabilitation, and Guangdong Key Laboratory of Mental Health and Cognitive Science, South China Normal University, Guangzhou, China

**Keywords:** parenting style, autistic traits, behavioral problem, autism spectrum disorder, children

## Abstract

**Background:**

Children with autism spectrum disorder (ASD) are at high risk of experiencing externalizing and internalizing problems. This study aimed to reveal how maternal parenting styles and autistic traits influence behavioral problems in children with ASD.

**Methods:**

This study recruited 70 2–5 years children with ASD and 98 typically developing (TD) children. The Parental Behavior Inventory (PBI) and Autism Spectrum Quotient (AQ) were used to collect the maternal parenting styles and autistic traits, respectively. The children’s behavioral problems were reported by the mothers using the Strengths and Difficulties Questionnaire (SDQ). Hierarchical moderated regression analyses were used to determine whether maternal autistic traits moderated the association between parenting style and behavioral problems in the children.

**Results:**

Compared to TD children, children with ASD exhibited more severe externalizing and internalizing problems (*t* = 4.85, *p* < 0.01). The ASD group scored lower in the maternal supportive/engaged parenting style than the TD group (*t* = 3.20, *p* < 0.01). In the TD group, the maternal AQ attention switching domain was positively correlated with internalizing problems in the children (*β* = 0.30, *p* = 0.03). In the ASD group, hostile/coercive parenting style was significantly correlated with externalizing problems in the children (*β* = 0.30, *p* = 0.02), whereas maternal AQ attention switching domain was negatively correlated with externalizing problems (*β* = −0.35, *p* = 0.02). Moreover, the maternal AQ attention switching domain moderated the association between hostile/coercive parenting style and children’s externalizing problems (*β* = 0.33, *p* = 0.04).

**Conclusion:**

Among ASD children, a hostile/coercive parenting style can increase the risks of children’s externalizing problems, especially in the context of high levels of maternal attention-switching problems. Hence, the current study has important implications for the clinical practice of early family-level interventions for children with ASD.

## Introduction

Autism spectrum disorder (ASD) is a group of neurodevelopmental disorders with different levels of social communication difficulties as well as the presence of restricted interests or stereotyped behaviors (1). Currently, ASD affects 1 in 44 children in the United States (2). Compared to typically developing (TD) children or children with other disabilities (3, 4), children with ASD are more likely to exhibit comorbid high levels of internalizing (e.g., depression or anxiety symptoms) and externalizing (e.g., hyperactivity, conduct problems, and attentional control) problems. Literature showed that the incidences of behavioral/conduct problems, anxiety, and depression in children with ASD are 60.8, 39.5, and 15.7%, respectively (3). Given that high rates of comorbid internalizing and externalizing problems in early childhood have a long-term impact on individuals, families, and society (5), the factors that may influence these problems in children with ASD need to be fully understood.

Parenting style is defined as a collection of parents’ attitudes, behaviors, and emotions (6). The two-factor theory (7, 8) suggests a reciprocal relationship in which parenting behaviors actively shape child behaviors as well as the other way around. For example, one study suggested that positive parenting styles reduced the risk of emotional and behavioral problems in TD children (9). By contrast, Pinquart et al. (10) found that permissive and neglectful parenting styles were more closely related to higher levels of externalizing problems. In children with ASD, previous studies also showed that less affection, more overprotection, and authoritarian control from the parents were significantly correlated to more severe behavioral problems (11, 12). These studies indicated that parenting style may be an important factor in the behavioral problems of children. However, the relations between parenting style and child’s behavioral problems have been mixed. In contrast, previous studies have shown non-directional (13) or bidirectional (14) relations between parenting style and behavioral problems in TD children. One of the theoretical mechanisms underlying the association between parenting style and behavior problems in children is the coercion theory (15). The central idea is that a low level of support and neglectful parenting styles as risk factors for children’s behavioral problems, and negative reinforcement will lead to the persistence of bad behavior of children and parents (10). Unfortunately, whether or not the different dimensions of parenting style lead to different behavioral problems in children with ASD and TD remains unclear. Thus, determining the direction of parenting styles as it relates to a child’s behavioral problems may help guide parents in their approach to parenting a child with ASD.

Literature showed that parental characteristics may be related to parenting styles (16). Autistic traits fall below the threshold of clinical significance in ASD characteristics that are similar to but are not as severe as those with a clinical diagnosis of ASD (17, 18). There are several dimensions of autistic traits, such as the Autism Spectrum Quotient (AQ), which includes social skills, attention shift, attention to detail, communication, and imagination (19). Many studies showed that compared to parents of TD children, the parents of children with ASD had significantly higher levels of autistic traits than controls (20–22). Currently, several studies indicated that parental autistic traits were not only correlated with autism severity (23, 24) and children’s self-regulation (25) but also with childhood social–emotional behavior in children (26). A review suggested that parental autistic traits may be important in parsing heterogeneity in ASD etiology (27) and in developing parent-mediated ASD interventions (28). However, few studies have been conducted to investigate the association between the domains of parental autistic traits and behavioral problems in children with ASD.

In addition, some studies have tried to explore the relationships between parental autistic traits and negative aspects of parenting. For example, a few studies showed that higher parental autistic traits are associated with more parenting difficulties (29), lowest parenting efficacy (30), and poorer mental health (31). Some study suggested that poor attention switching (domain of autistic traits) was associated with parenting difficulties (32), and the communication of maternal autism traits in children with ASD was positively correlated with problem-solving of family function (15, 33).

Therefore, we hypothesize that some domains of autistic traits may be related to the parenting style and child behaviors and, thus, potentially moderate the relationship between the two in children with ASD. As noted, the previous study suggested differences in maternal and paternal parenting styles (34). Mothers spend more time with their children and have more caregiving and managerial roles compared to fathers (35–37). Thus, our study focused on the influence of maternal rearing patterns and autistic traits on behavioral problems in children. The current study may help adjust maternal parenting styles according to autistic traits and may provide a basis for early family intervention of ASD children.

In summary, the study aims to first, examine the differences in the behavioral problems of children with ASD and those with TD; second, evaluate the differences in maternal parenting styles between ASD and TD groups and determine whether a relationship exists between parenting styles and behavioral problems in the children; and finally, determine whether maternal autistic traits moderate the association between parenting styles and behavioral problems in the children.

## Method

### Participants and procedures

In this cross-sectional study, 70 children with ASD (Mage = 3.52, SD = 0.98), 98 TD children (Mage = 3.07, SD = 0.92), and all their mothers were recruited in Foshan, China, between May 2021 and September 2022. The inclusion criteria of the ASD group were diagnosed as ASD by two chief psychiatrists (SJH and HY) using the Diagnostic and Statistical Manual of Mental Disorders, Fifth Edition (DSM-5) criteria, and the total score in the Children Autism Rating Scale (CARS) of all children with ASD was a score above 36. A group of TD children without ASD diagnosis was also recruited from the same child healthcare clinic. Those who had a history of developmental delay were excluded from the TD group. Additional inclusion criteria for selecting both groups were chronological age (between 2 and 5 years old) and all the children accompanied by mothers. Known genetic or chromosomal abnormalities (such as down syndrome and fragile X syndrome), significant organic diseases (such as blindness and deafness), and comorbidity with other mental disorders (such as childhood schizophrenia and attention-deficit hyperactivity disorder) based on the self-report by mothers were excluded for selecting both groups.

First, the selecting ASD group was recruited after the diagnosis by the doctor and the CARS score was greater than 36. Then, all the children accompanied by their mothers were invited to participate in this study. All mothers provided informed consent before they were individually interviewed by health personnel who received standardized training in conducting face-to-face interviews. Mothers of the two groups were asked to answer many structured questionnaires on behavioral problems in the children as well as in maternal parenting styles and autism-like traits, among others. This study was approved by the Ethics Committee of the School of Public Health at Sun Yat-sen University.

### Demographic information

Using self-made questionnaires, we collected the demographic information of the subjects, including maternal age, maternal education, income, child age, and child gender.

### Strengths and Difficulties Questionnaire (SDQ)

The Strengths and Difficulties Questionnaire ((SDQ)-parent form) (38) was adapted by Kou and Du from Goodman et al.’s study (39). The SDQ is a 25-item that uses a 3-point Likert scale (“not true,” “somewhat true,” or “certainly true”) and is designed to assess behavioral and emotional problems in children and adolescents ages 3–17 (38). Previous studies also show that the scale is applicable to 2-year-old children in China (40). Thus, mothers were asked to complete the extended parent version of the SDQ for ASD children. The items are divided into five subscales: hyperactivity/inattention, emotional symptoms, peer problems, conduct problems, and prosocial behavior. The composite scores for internalizing problems (emotional and peer items) and externalizing problems (conduct and hyperactivity items) were then analyzed in the current study (41). The SDQ has good reliability and structural validity in Chinese individuals (38).

### Parental Behavior Inventory (PBI)

The PBI is a 20-item, parent-report questionnaire that uses a 6-point Likert scale *(never to always)* (42). The questionnaire yields two subscales: support/participation and hostility/coercion parenting styles. It is used to assess the parenting style among preschool and school-age children. Mothers were asked to complete the questionnaire. PBI has good reliability and structural validity in Chinese populations, with Cronbach’s α values of 0.807 and 0.652 for support/participation and hostility/coercion, respectively, for Chinese individuals (43).

### Autism Spectrum Quotient (AQ) in adults

Maternal autistic traits were evaluated using the AQ (19). The AQ comprises 50 questions and uses a 4-point Likert scale *(absolutely agree to absolutely disagree)*, with 10 questions assessing five different domains: social skills, attention switching, attention to detail, communication skills, and imagination. Each of these items scores 1 point if the respondent records the autistic-like trait either mildly or strongly. The maximum value for each subscale is 10 points. A higher score indicates more severe autistic traits. The AQ has good reliability and structural validity in Chinese adults (44).

### Children Autism Rating Scale (CARS)

This scale is used by trained professionals to assess the interpersonal relationship, imitation, emotional response, language communication, and other aspects of autistic children over a period of 18 months. The scale is composed of 15 items that use a 4-point Likert scale (age-related performance, mild abnormality, moderate abnormality, and severe abnormality). The higher the total score on the scale, the more serious the symptoms; a total score of between 30 and 36 indicates mild–moderate autism, and a score above 36 indicates severe autism (45). The Cronbach’s alpha coefficient is 0.73 in the Chinese version (46). In the current study, autistic children with a score above 36 were included.

### Gesell Development Schedules (GDS)

The GDS is currently a widely used neurodevelopmental scale that consists of five domains, namely adaptability, gross motor, fine motor, language, and personal-social (47). The developmental quotient (DQ) of the five domains is used to evaluate the level of neurodevelopment. The higher the DQ, the better the neurodevelopment. The GDS has good reliability and validity in Chinese children (48). In the current study, a GDS score greater than 85 in all children is considered normal.

### Statistical analysis

SPSS v23.0 statistical software was used for the statistical analysis. Descriptive statistics for continuous variables are presented as the mean (M) and standard deviation (SD), and count data are described by prevalence (%). Differences in demographic information and behavioral outcomes between the ASD and TD groups were compared by the *t*-test or chi-square test, with a significance level of *p* < 0.05. Hierarchical moderated regression analyses were used to determine whether or not maternal autistic traits moderate the association between maternal parenting styles and behavioral problems in the children. In the first step of the regression models, the independent variables were maternal parenting style (supportive/engaged vs. hostile/coercive) and maternal AQ domains separately across groups, controlling for child age, gender, DQ, family income, family income meeting demand, and maternal education level. The second step of the regression models included interaction terms between different parenting styles and each AQ domain. All five interaction terms were included in each regression model. For all moderated regression analyses, standardized variables (*Z*-scores) were used to compute the product terms. Simple slope analysis was conducted using the Process 2.16 macro plug-in of SPSS 23.0. Standardized regression coefficients were presented on all betas, and the significance level was *p* < 0.05.

## Results

### Demographic information analysis

Compared to the TD group, the ASD group was significantly older (*t* = −3.08, *p* < 0.01) and had significantly lower DQ (*t* = 23.13, *p* < 0.01) and maternal education levels (χ^2^ = 30.88, *p* < 0.01). No significant differences were found in the maternal age, child gender, or family income (*ps* > 0.05) of the two groups. The results are shown in Table 1.

**Table 1:** Descriptive statistics for autism and control groups.

Variables		ASD (*n* = 70)	TD (*n* = 98)	χ^2^/t (*p* value)
		Mean (SD)/n (%)	Mean (SD)/n (%)	
Gender	Boys	55 (78.6%)	70 (71.4%)	1.09 (0.30)
	Girls	15 (21.4%)	28 (28.6%)	
Age (years)		3.52 (0.98)	3.07 (0.92)	**−3.08 (*p* < 0.01)**
DQ		57.04 (10.97)	102.88 (13.73)	**23.13 (*p* < 0.01)**
Maternal age (years)		33.31 (4.99)	32.61 (4.21)	−0.99 (0.32)
**Family income**				
	<￥8,000	41 (58.6%)	65 (66.3%)	1.05 (0.30)
	≥￥8,000	29 (41.4%)	33 (33.7%)	
Family income meeting demand				
	No	13 (18.6%)	5 (5.1%)	**7.74 (0.005)**
	Yes	57 (81.4%)	93 (94.9%)	
**Maternal education**				
	Low (primary, secondary, high school, and uneducated)	35 (50.0%)	11 (11.2%)	**30.88 (*p* < 0.01)**
	High (university and above)	35 (50.0%)	87 (88.8%)	
SDQ				
	Child externalizing problems	8.61 (2.35)	6.36 (3.05)	**4.09 (<0.01)**
	Child internalizing problems	7.30 (2.45)	4.93 (2.47)	**4.85 (<0.01)**
**Parenting style**				
	Supportive/engaged	34.84 (9.84)	38.82 (6.23)	**3.20 (<0.01)**
	Hostile/coercive	16.66 (7.03)	18.01 (7.17)	1.21 (0.61)
**Maternal AQ domains**				
	Social skill	3.56 (2.56)	3.89 (2.64)	0.81 (0.42)
	Attention switching	4.03 (1.79)	4.40 (1.79)	−0.37 (0.71)
	Attention to detail	4.74 (2.31)	4.61 (2.21)	0.17 (0.87)
	Communication	2.84 (2.28)	2.90 (2.03)	−1.34 (0.18)
	Imagination	3.53 (1.44)	3.21 (1.55)	0.33 (0.75)

ASD, autism spectrum disorder; TD, typical development; DQ, developmental quotient; SDQ, Strengths and Difficulties Questionnaire; AQ, Autism Spectrum Quotient. The bold values, statistically significant.

### Comparison of children’s behavioral problems, parenting style, and maternal autistic traits of the two groups

The children with ASD exhibited significantly more externalizing problems (*t* = 4.09, *p* < 0.01) and internalizing problems (*t* = 4.85, *p* < 0.01) than the TD children. Moreover, compared to the mothers of the TD children, those of children with ASD scored lower in supportive/engaged parenting style (*t* = 3.20, *p* < 0.01). However, no significant differences were found in the maternal AQ domains of the ASD and TD groups (*ps >* 0.05). The results are shown in Table 1.

### Association between maternal parenting style and autistic traits and child behavioral problems

Multiple regression analysis showed that hostile/coercive parenting style significantly increased externalizing problems (*β* = 0.30, *p* = 0.02) only in the children in the ASD group after controlling for child age, gender, DQ, family income, family income meeting demand, maternal age, and maternal education level. In addition, the attention switching domain of maternal AQ was negatively correlated with externalizing problems (*β* = −0.35, *p* = 0.02) in ASD children. In the TD group, the maternal AQ attention switching domain was negatively associated with internalizing problems in the children (*β* = −0.30, *p* = 0.03). The results are shown in Tables 2, 3.

**Table 2:** Moderated multiple regression model of the hostile/coercive parenting behavior on behavior problems in children with ASD and TD.

Independent variables	ASD group (*n* = 70)						TD group (*n* = 98)					
	Externalizing problems			Internalizing problems			Externalizing problems			Internalizing problems		
	Adjusted β	*b (SE)*	*p*	Adjusted β	*b (SE)*	*p*	Adjusted β	*b (SE)*	*p*	Adjusted β	*b (SE)*	*p*
Step one *R*^2^	0.18*			0.07			**0.15** *			0.04		
Hostile/coercive	**0.30**	**0.04**	**0.02**	0.21	0.05	0.12	0.14	0.05	0.24	0.17	0.04	0.18
**AQ domains**												
Social skill	0.05	0.13	0.73	−0.12	0.16	0.43	−0.05	0.14	0.72	−0.13	0.13	0.37
Attention switching	**−0.35**	**0.18**	**0.02**	0.10	0.22	0.50	0.08	0.20	0.53	**0.30**	**0.18**	**0.03**
Attention to detail	−0.13	0.12	0.28	−0.08	0.15	0.54	0.13	0.13	0.22	0.02	0.12	0.87
Communication	−0.01	0.14	0.96	0.09	0.18	0.56	−0.04	0.17	0.75	0.00	0.16	1.00
Imagination	−0.05	0.22	0.69	−0.12	0.16	0.43	−0.05	0.14	0.72	−0.01	0.18	0.95
Step two *R*^2^	0.18*			0.13			**0.25** *			−0.01		
Hostile/coercive	**0.45**	**0.05**	**0.00**	0.16	0.06	0.28	0.23	0.05	0.08	0.12	0.05	0.45
AQ domains												
Social skill	0.00	0.13	1.00	−0.14	0.16	0.35	−0.01	0.16	0.92	−0.16	0.16	0.33
Attention switching	−0.25	0.19	0.09	0.06	0.23	0.67	0.02	0.19	0.86	**0.32**	**0.20**	**0.03**
Attention to detail	−0.11	0.13	0.42	−0.08	0.16	0.56	0.08	0.13	0.44	0.01	0.13	0.92
Communication	−0.06	0.15	0.66	0.15	0.18	0.34	−0.05	0.16	0.65	0.02	0.16	0.90
Imagination	−0.09	0.22	0.51	−0.02	0.27	0.89	0.12	0.20	0.31	−0.01	0.21	0.92
**Moderating role of AQ domains**												
Hostile/coercive ×Social skill	−0.11	0.02	0.55	0.27	0.03	0.17	−0.18	0.02	0.30	0.13	0.02	0.52
Hostile/coercive ×Attention switching	**0.33**	**0.03**	**0.04**	−0.04	0.03	0.79	0.19	0.03	0.19	0.07	0.03	0.66
Hostile/coercive ×Attention to detail	−0.04	0.02	0.82	0.09	0.03	0.58	**0.35**	**0.02**	**0.00**	−0.01	0.02	0.96
Hostile/coercive ×Communication	0.05	0.02	0.73	−0.21	0.03	0.18	−0.06	0.03	0.67	−0.16	0.03	0.36
Hostile/coercive ×Imagination	−0.14	0.04	0.25	**0.34**	**0.04**	**0.01**	0.10	0.03	0.41	−0.03	0.03	0.84

*p ≤ 0.05. Adjusted variable child’s gender, age, DQ, family income, family income meeting demand, and maternal education. ASD, autism spectrum disorder; TD, typical development; DQ, developmental quotient; SDQ, Strengths and Difficulties Questionnaire; AQ, Autism Spectrum Quotient. The bold values, statistically significant.

**Table 3:** Moderated multiple regression model of the supportive/engaged parenting behavior on behavior problems in children with ASD and TD.

Independent variables	ASD group (*n* = 70)						TD group (*n* = 98)					
	Externalizing problems			Internalizing problems			Externalizing problems			Internalizing problems		
	Adjusted β	*b (SE)*	*p*	Adjusted β	*b (SE)*	*p*	Adjusted β	*b (SE)*	*p*	Adjusted β	*b (SE)*	*p*
Step one *R*^2^	0.09			0.03			**0.14** *			0.02		
Supportive/engaged	0.02	0.03	0.86	0.03	0.04	0.85	−0.03	0.05	0.80	−0.02	0.05	0.87
**AQ domains**												
Social skill	0.15	0.13	0.32	−0.06	0.16	0.71	−0.05	0.14	0.72	−0.13	0.13	0.34
Attention switching	**−0.33**	**0.19**	**0.03**	0.11	0.23	0.45	0.10	0.20	0.43	**0.33**	**0.18**	**0.02**
Attention to detail	−0.16	0.13	0.22	−0.10	0.16	0.47	0.13	0.13	0.23	0.02	0.12	0.87
Communication	−0.07	0.15	0.63	0.05	0.19	0.76	−0.03	0.17	0.83	0.02	0.16	0.90
Imagination	−0.07	0.23	0.63	−0.07	0.29	0.63	0.08	0.20	0.47	−0.01	0.18	0.96
Step two R^2^	0.10			**0.20** *			0.097			0.03		
Supportive/engaged	−0.03	0.03	0.84	0.17	0.04	0.19	−0.03	0.05	0.81	−0.05	0.05	0.68
**AQ domains**												
Social skill	0.18	0.13	0.22	−0.09	0.15	0.53	0.02	0.18	0.91	−0.14	0.16	0.43
Attention switching	**−0.36**	**0.19**	**0.02**	0.21	0.22	0.14	0.13	0.25	0.43	**0.42**	**0.23**	**0.02**
Attention to detail	−0.17	0.13	0.20	−0.09	0.14	0.44	0.17	0.17	0.19	0.18	0.15	0.20
Communication	−0.02	0.16	0.91	−0.07	0.18	0.64	−0.07	0.27	0.71	0.10	0.24	0.64
Imagination	−0.05	0.25	0.74	−0.10	0.28	0.49	0.05	0.25	0.75	−0.14	0.23	0.34
**Moderating role of AQ domains**												
Supportive/engaged ×Social skill	−0.38	0.02	0.054	**0.64**	**0.02**	**0.00**	−0.12	0.03	0.50	0.03	0.02	0.87
Supportive/engaged ×Attention switching	0.02	0.02	0.90	−0.03	0.03	0.86	−0.03	0.03	0.87	−0.08	0.03	0.64
Supportive/engaged ×Attention to detail	−0.07	0.01	0.62	−0.10	0.02	0.43	−0.07	0.02	0.63	−0.28	0.02	0.06
Supportive/engaged ×Communication	0.41	0.02	0.06	**−0.59**	**0.02**	**0.01**	0.07	0.03	0.71	−0.13	0.03	0.54
Supportive/engaged ×Imagination	−0.08	0.02	0.58	0.00	0.03	1.00	0.08	0.04	0.55	0.23	0.03	0.11

*p ≤ 0.05. Adjusted variables: child’s gender, age, DQ, family income, family income meeting demand, and maternal education. ASD, autism spectrum disorder; TD, typical development; DQ, developmental quotient; SDQ, Strengths and Difficulties Questionnaire; AQ, Autism Spectrum Quotient. The bold values, statistically significant.

### Moderation of maternal autistic traits on the relationship between maternal parenting style and children’s behavioral problems

In the moderation model, the main effect of maternal hostile/coercive parenting style on externalizing problems in the children remained significant (*β* = 0.45, *p* < 0.01). Moreover, this association was moderated by maternal attention switching (*β* = 0.33, *p* = 0.04) in the ASD group. In the model, we controlled for child age, gender, DQ, family income, family income meeting demand, maternal age, and maternal education level. The results are shown in Tables 2, 3.

**Figure 1:**
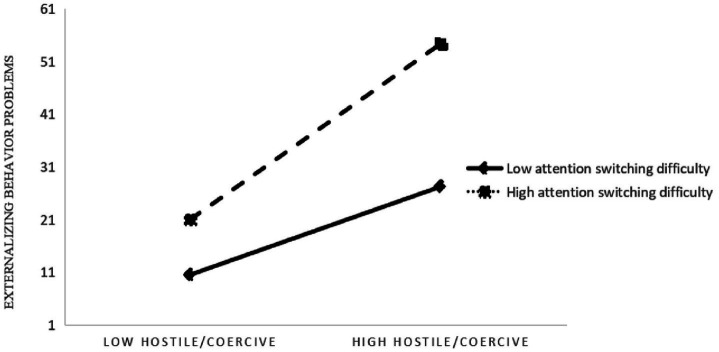
Maternal attention switching moderating the association between hostile/coercive parenting style and externalizing problems in children with ASD. At high levels of maternal attention switching problems, such that a greater use of hostile/coercive parenting style was associated with more externalizing problems in the children (*p* < 0.01). By contrast, at low levels of attention switching problems, the correlation between maternal hostile/coercive parenting style and externalizing problems was non-significant (*p* = 0.15). ASD, autism spectrum disorder.

More importantly, the significant interaction between hostile/coercive parenting style and maternal AQ attention switching domain for child externalizing problems was probed and plotted at one standard deviation above and below the mean of the moderator (AQ attention switching severity; see Figure 1). Simple slope tests demonstrated that the association between maternal hostile/coercive parenting style and externalizing problems in the children with ASD was significant at high levels of maternal attention switching problems, such that greater use of hostile/coercive parenting style was associated with more externalizing problems in the children (*b* = 0.22, *t* = 3.43, *p* < 0.01). Similar results were found at medium levels of maternal attention switching problems (*b* = 0.14, *t* = 3.64, *p* < 0.01). By contrast, at low levels of attention switching problems, the correlation between maternal hostile/coercive parenting style and externalizing problems was non-significant (*b* = 0.06, *t* = 1.46, *p* = 0.15; Figure 1).

## Discussion

The findings of this study are as follows: (1) The children with ASD had more severe externalizing and internalizing problems than the TD children, and the maternal supportive/engaged parenting style scores in the ASD group were lower than those in the TD group; (2) maternal hostile/coercive parenting style was positively associated with child externalizing problems, and maternal AQ attention switching domain was negatively correlated with child externalizing problems in the ASD group; and (3) maternal AQ attention switching domain moderated the association between hostile/coercive parenting style and externalizing problems in children with ASD.

### Association between maternal parenting style and behavioral problems in children with ASD

The current study showed that children with ASD exhibited more serious externalizing and internalizing problems than TD children. Consistent with our results, the literature showed that children with ASD had more severe internalizing problems such as anxiety/depressive symptoms than their TD peers (49–51). Moreover, ASD children displayed more externalizing problems such as peer conflict (49) and attention-deficit and hyperactivity symptoms (52, 53). We also found that the ASD group scored lower in maternal supportive/engaged parenting style than the TD group. Consistent with our findings, the literature indicated that children with ASD received less maternal affection and warmth than TD children (11, 54). It is possible that children with ASD lack reciprocal relationships, have communication impairments, and decreased response to social stimulation, which may result in a decrease in parents’ affectionate interactions with these children (55, 56). Of course, some studies showed that no significant differences were found in the maternal parenting styles in the ASD and TD groups (57, 58). This inconsistency may be the sample size, sample type, or limitation of the self-report instrument. Further studies are needed to determine the reasons for differing results.

Notably, the current study showed that maternal hostile/coercive parenting style was associated with externalizing behaviors in children with ASD. Similar to our results, previous studies suggested that children with ASD aged 6–10 years exhibited more externalizing behaviors under conditions of higher levels of parental critical comments and harsh-discipline parenting style (59), and higher levels of maternal discipline and harsh punishment parenting style were related to greater externalizing problems in ASD (12, 60). According to the coercion theory (15), we speculate that raising a child with ASD is quite stressful for parents because of the child’s social communication difficulties, narrow interests or stereotyped behaviors, and internalizing and externalizing behaviors, which can lead to hostile/coercive parenting styles. In turn, such a negative parenting style can increase the frequency and intensity of sadness, anger, and bad behaviors further in children with ASD (15). Thus, the relationship between hostile/coercive parenting and externalizing behavior highlights the importance of interventions that may break this negative cycle. By contrast, maternal hostile/coercive parenting style was not related to externalizing behaviors in TD children. The reason is not clear, and we suspect that it may be due to autistic traits in children with ASD such as persistent difficulties in social communication and interaction (1) and deficits in emotional functioning (49).

### Moderation of maternal autistic traits in the relationship between maternal parenting style and externalizing behavioral problems in children with ASD

Attention switching is defined as diverting attention between tasks, which is considered a core cognitive ability that underlies the executive control of thought and action (61). Attention switching problems are expressed that people frequently get so strongly absorbed in one thing that loses sight of other things (19). Our multilevel analyses indicated that maternal attention switching was negatively associated with externalizing problems in the ASD group. We speculated that maternal autistic traits may affect children’s behavior either as a genetic predisposition (62) or together with other factors such as parenting style.

In the current study, we found that more likely hostile/coercive parenting styles were associated with more children’s externalizing problems at medium and high levels of maternal attention switching problems. Neuropsychological testing shows that parents of children with ASD have impaired executive function skills in the area of attentional flexibility (63). Moreover, previous evidence suggested that parents of children with ASD were found to have a “generativity deficit” in patterns meaning tasks (64). As mentioned previously, mothers may have a more negative parenting style (32) and potentially further impact parent–child interactions (65) in the context of high attention switching difficulty. Similarly, mothers with attention conversion difficulties may affect the development of children’s emotional and behavioral regulation abilities (25). One of the possible explanations for the moderate effect of attention switching difficulty on the association between hostile/coercive parenting style and children’s externalizing problems is that children’s needs and behaviors will change with different situations (66); however, the mother with poor attention switching will lack flexibility in parenting style, resulting in more hostility or coercion (32, 67). Based on the coercion theory (15), a more hostile/coercive parenting style may affect the interaction with the child and impact the child’s social–emotional engagement, communication, and social interaction abilities (68, 69), which then leads to poorer child developmental outcomes. Thus, maternal parenting style and autistic traits should be assessed early and should be included in early family-level interventions to help mothers to develop strategies for interaction with ASD children.

Several limitations of the study should be acknowledged. First, our study design was cross-sectional and thus could not prove the causal relationship between maternal parenting style and autistic traits with behavioral problems in the children. Second, this study only included female caregivers. Further research is needed to clarify the influence of both maternal and paternal characteristics on children’s developing behavioral problems. Third, all indicators in the study were assessed by a questionnaire. In future, cognitive assessments can be used to improve the objectivity of the results. Fourth, only children with severe autism were selected for the current study, and future studies will be considered the effect of the severity of autism on parenting style. Finally, the sample size is small; thus, future studies should consider these questions in a larger sample.

## Conclusion

Children with ASD exhibited more severe externalizing and internalizing problems than TD children. Moreover, the ASD group scored lower in maternal supportive/engaged parenting style scores than the TD group. Among mothers with greater use of hostile/coercive, their autistic children were more likely to present externalizing problems. In particular, the maternal AQ attention switching domain moderated the association between hostile/coercive parenting styles and externalizing problems in children with ASD. The current study suggests mothers of children with ASD reduce hostile/coercive parenting styles and improve their maternal attention switching may reduce externalizing problems.

## Data availability statement

The raw data supporting the conclusions of this article will be made available by the authors, without undue reservation.

## Ethics statement

The study was conducted according to the guidelines of the Declaration of Helsinki, and approved by the Ethics Committee of the School of Public Health at Sun Yat-sen University. The code is 20210124. Written informed consent to participate in this study was provided by the participants’ legal guardian/next of kin.

## Author contributions

XjL designed the study. XS, SH, HY, and XjL performed the survey research. LL, MC, and ZL analyzed the data. XjL drafted the manuscript. XW, XhL, and JJ were the critical revision of the manuscript for important intellectual content. All authors contributed to the article and approved the submitted version.

## Funding

This research was supported by the Key R&D Program of Guangdong Province (grant number 2019B030335001), the Social-Area Science and Technology Research Program of Foshan City (Department of Science and Technology of Foshan City, 2120001008276), and the National Natural Science Foundation of China (grant number 81872639).

## Conflict of interest

The authors declare that the research was conducted in the absence of any commercial or financial relationships that could be construed as a potential conflict of interest.

## Publisher’s note

All claims expressed in this article are solely those of the authors and do not necessarily represent those of their affiliated organizations, or those of the publisher, the editors and the reviewers. Any product that may be evaluated in this article, or claim that may be made by its manufacturer, is not guaranteed or endorsed by the publisher.
